# Developmental Defects of Enamel in the Pediatric Population Attending a Tertiary Care Hospital in Kashmir: A Hospital-Based Prevalence Study

**DOI:** 10.7759/cureus.112736

**Published:** 2026-07-15

**Authors:** Insha Showkat, Nazia Lone, Sana Farooq, Nadia Irshad, Mohsin Sidiq, Waseem R Nadaf

**Affiliations:** 1 Pediatric Dentistry, Government Dental College and Hospital, Srinagar, IND

**Keywords:** demarcated opacity, developmental defects of enamel, diffuse opacity, enamel hypoplasia, kashmiri population, pediatric dentistry

## Abstract

Background

Developmental defects of enamel (DDE) are disturbances that occur during amelogenesis and may affect the quantity or quality of enamel. These defects may predispose affected teeth to dental sensitivity, caries, and esthetic concerns. Limited epidemiological data are available regarding the prevalence of DDE among Kashmiri children.

Objective

This study aims to evaluate the prevalence and distribution of DDE among children aged 3-12 years in the Kashmiri population using the modified DDE Index.

Methods

A hospital-based cross-sectional epidemiological study was conducted among children aged 3-12 years attending the outpatient department (OPD) of Pediatric and Preventive Dentistry at Government Dental College and Hospital, Srinagar, India. A total of 10,070 children were examined over a period of seven months. The modified DDE Index was used to assess enamel defects in both primary and permanent dentition. Buccal/labial, lingual/palatal, and occlusal/incisal surfaces were examined. Demographic variables, including age group as well as dentition type (primary and permanent), were recorded to evaluate their association with the prevalence and distribution of enamel defects. Data were analyzed using Python version 3.8 (Python Software Foundation, Fredericksburg, VA), and a chi-square test was applied for statistical analysis. Chi-square test of independence demonstrated a statistically significant association between DDE prevalence and dentition type (primary and permanent dentition) (p = 0.0001).

Results

Among the 10,070 children examined, 1,208 children demonstrated DDE involving 3,308 teeth. The prevalence of DDE was highest in the 9-12-year age group (2570, 77.69%), followed by the 6-9-year age group (696, 21.03%) and the 3-6-year age group (42, 1.2%). Permanent incisors were the most commonly affected teeth (2162, 65.35%), followed by permanent molars (754, 22.79%). Demarcated opacities (DEO) were the most prevalent enamel defect (816, 49.28%), followed by hypoplasia (408, 24.64%) and diffuse opacities (DIO) (324, 19.57%). Chi-square analysis demonstrated a statistically significant association between age groups (3-6, 6-9, and 9-12 years) and the distribution of DDE (p < 0.05). It also revealed a statistically significant variation in the distribution of developmental enamel defects between primary and permanent dentition and across different categories of permanent teeth, including molars, canines, premolars, and incisors (χ² = 361.92, p < 0.0001).

Conclusion

DDE was predominantly observed in permanent incisors and molars among Kashmiri children, with DEO being the most common defect type. The findings highlight the need for preventive and early intervention strategies to reduce the burden of DDE in pediatric populations.

## Introduction

Developmental defects of enamel (DDE) are commonly encountered in the pediatric population and represent disturbances in the quality or quantity of enamel resulting from alterations during amelogenesis. The severity and clinical presentation of these defects depend on the stage of enamel formation at which the insult occurs, as well as the duration and intensity of the disturbance [1].

Enamel hypoplasia (EH) is considered a quantitative defect characterized by reduced enamel thickness, whereas enamel hypomineralization is a qualitative defect associated with altered enamel translucency and opacity. Hypomineralized lesions may present clinically as diffuse opacities (DIO) or demarcated opacities (DEO), appearing as white, yellow, or brown discolorations [2]. These defects may adversely affect esthetics, increase tooth sensitivity, predispose teeth to dental caries, and contribute to occlusal dysfunction.

Among the various developmental enamel defects, molar incisor hypomineralization (MIH) has received increasing attention because of its clinical and public health significance. MIH is a qualitative enamel defect affecting one or more first permanent molars, often accompanied by involvement of permanent incisors. Affected teeth may exhibit hypersensitivity, post-eruptive enamel breakdown, increased susceptibility to dental caries, and restorative difficulties. Clinically, MIH should be differentiated from other enamel defects such as EH, dental fluorosis, and amelogenesis imperfecta, as these conditions may present with similar enamel discolorations or structural changes but differ in etiology, distribution, and treatment requirements [3].

The etiology of DDE is complex and multifactorial, involving interactions between genetic predisposition and environmental influences. Potential risk factors include maternal illness during pregnancy, nutritional deficiencies, prematurity, low birth weight, birth complications, systemic diseases, prolonged childhood fevers, respiratory infections, and other disturbances occurring during critical periods of enamel formation. Given the diversity of contributing factors, the identification of a single causative pathway is often challenging [4].

Recent epidemiological studies have reported a substantial global burden of developmental enamel defects, emphasizing their growing relevance to pediatric oral health and preventive dentistry [5].

The modified DDE Index, adapted from the original DDE Index, is widely used in epidemiological and observational studies because of its reproducibility and standardized method of recording enamel defects. The index classifies defects into three major categories: DEO, DIO, and hypoplastic defects [6]. Additionally, the extent of the defect is recorded according to the proportion of the tooth surface involved [7].

Previous studies have demonstrated considerable variation in the prevalence and distribution of DDE among different populations and ethnic groups. However, there is limited published data regarding the prevalence of DDE among children in Kashmir. Therefore, the present study aimed to evaluate the prevalence of DDE among children aged 3-12 years in Kashmir using the modified DDE Index.

## Materials and methods

This hospital-based cross-sectional epidemiological study was conducted in the Department of Pediatric and Preventive Dentistry at Government Dental College and Hospital, Srinagar, India, over a period of seven months starting from June 1, 2024, to January 3, 2025. The study protocol was reviewed and approved by the Institutional Ethics Committee of Government Dental College and Hospital (Approval No.: GDC/Perio/Eth Committee/1804; dated July 24, 2024) and was conducted in accordance with the principles of the Declaration of Helsinki.

Study population

Children aged 3-12 years attending the outpatient department (OPD) of Pediatric and Preventive Dentistry for their first dental visit were included in the study. Written informed consent was obtained from the parents or guardians before participation.

Inclusion and exclusion criteria

Children aged 3-12 years who had not undergone any previous treatment for enamel defects were included in the study. Teeth with gross carious lesions involving the buccal/labial surfaces were excluded from the examination.

Sample size calculation

The sample size was estimated using the formula for prevalence studies:

n = Z²P(1 − P)/d²

where n is the required sample size, Z is the standard normal deviate corresponding to a 95% confidence level (1.96), P is the anticipated prevalence, and d is the desired precision. As no recent population-based estimate of DDE was available for the study population, a prevalence of 50% was assumed to provide the maximum sample size, with a precision of 5%. The minimum required sample size was calculated to be 384 children. During the study period, all eligible children aged 3-12 years attending the OPD for their first dental visit were consecutively recruited, resulting in a final sample of 10,070 children, which substantially exceeded the minimum required sample size and improved the precision of the prevalence estimates.

Clinical examination

Prior to clinical examination, oral prophylaxis was performed for all participants. A total of 10,070 children were evaluated during the study period. Clinical examination included assessment of all primary and permanent teeth. Three tooth surfaces - buccal, occlusal/incisal, and lingual/palatal - were examined under adequate illumination. To minimize examiner-related variability, three examiners were trained and calibrated prior to data collection, and standardized diagnostic criteria were used throughout the study. DDE was diagnosed and classified using the modified DDE Index [8]. The enamel defects were categorized into EH, DIO, and DEO.

Data collection and statistical analysis

A structured proforma was used to record demographic details, including age and gender, along with the modified DDE Index findings. The collected data were entered into Microsoft Excel 2016 (Microsoft Corp., Redmond, WA) and analyzed using Python (Python Software Foundation, Fredericksburg, VA).

**Figure 1:**
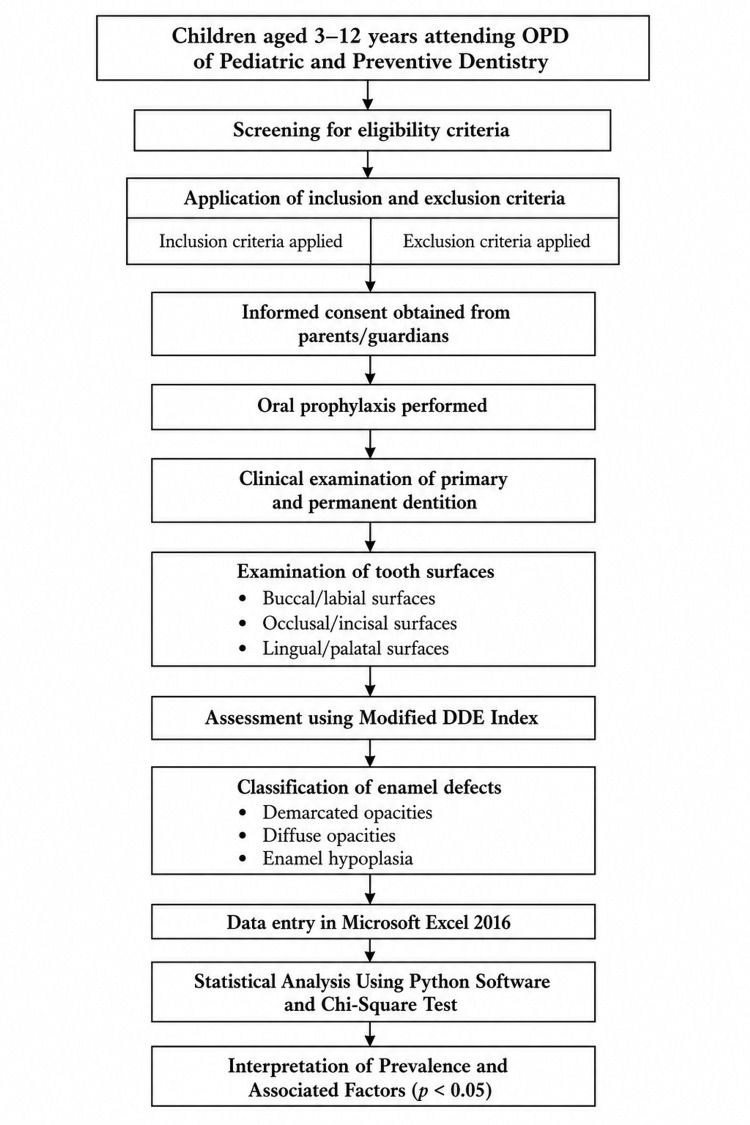
Flowchart of methodology OPD: Outpatient department; DDE: Developmental defects of enamel.

Descriptive and inferential statistical analyses were performed. The chi-square test was used to assess associations between variables, and a p-value of <0.05 was considered statistically significant. The methodology has been discussed in the form of a flowchart in Figure 1.

## Results

A total of 10,070 children aged 3-12 years were examined during the study period. DDE was identified in 3,308 (100%) teeth among 1,208 children. The distribution of DDE among different dentitions demonstrated that primary teeth accounted for 164 (4.95%) of the affected teeth, whereas permanent molars constituted 754 (22.79%) of the affected teeth (Table 1).

**Table 1:** Tooth-wise distribution of developmental defects of enamel among affected teeth

Tooth Type	Frequency (n)	Percentage (%)
Primary teeth	164	4.95
Permanent molars	754	22.79
Permanent canines	96	2.9
Permanent premolars	132	3.99
Permanent incisors	2162	65.35
Total	3308	100

Analysis according to age groups revealed that among the 3,308 affected teeth, 42 (1.2%) were observed in children aged 3-6 years, 696 (21.03%) in children aged 6-9 years, and 2570 (77.69%) in children aged 9-12 years (Figure 1).

In the 3-6-year age group, primary teeth represented 18 (42.8%) of the affected teeth, while permanent molars and permanent incisors each accounted for 12 (14.28%). In the 6-9-year age group, 78 (11.2%) of affected teeth were primary teeth, 462 (66.3%) were permanent incisors, 144 (20.68%) were permanent molars, and 6 (0.86%) each were permanent premolars and permanent canines. In the 9-12-year age group, 68 (2.64%) of affected teeth were primary teeth, 1694 (65.91%) were permanent incisors, 604 (23.5%) were permanent molars, 114 (4.43%) were permanent premolars, and 90 (3.5%) were permanent canines (Table 2).

**Table 2:** Distribution of developmental defects of enamel affecting various teeth according to age groups

Age Group	Primary Teeth, n (%)	Permanent Molars, n (%)	Permanent Canines, n (%)	Permanent Premolars, n (%)	Permanent Incisors, n (%)	Total, n (%)
3–6 years	18 (42.8)	12 (14.28)	0 (0.0)	0 (0.0)	12 (14.28)	42 (1.2)
6–9 years	78 (11.20)	144 (20.68)	6 (0.86)	6 (0.86)	462 (66.3)	696 (21.03)
9–12 years	68 (2.64)	604 (23.50)	90 (3.50)	114 (4.43)	1694 (65.91)	2570 (77.69)
Total	164 (4.95)	754 (22.79)	96 (2.90)	132 (3.99)	2162 (65.35)	3308 (100)
Chi-square test value = 361.92, p = 0.0001* (significant association)						

The prevalence of DDE was found to be 68 (0.67%) in primary teeth and 1160 (11.5%) in permanent teeth (Table 3). Among the 1,656 (100%) enamel defects identified, DEO was the most common defect type, accounting for 816 (49.28%) of cases. Hypoplastic defects constituted 408 (24.64%), while DIO accounted for 324 (19.57%). Combined defects were less frequently observed, with DEO associated with hypoplasia accounting for 90 (5.43%), DEO and DIO together accounting for 6 (0.36%), and DIO associated with hypoplasia also accounting for 6 (0.36%) (Table 4).

**Table 3:** Overall prevalence of developmental defects of enamel in primary and permanent dentition DDE: Developmental defects of enamel.

Dentition Type	DDE Cases (n/N)	DDE Prevalence (%)
Primary teeth	68/10070	0.67
Permanent teeth	1160/10070	11.5

**Table 4:** Percentage distribution of various enamel defects observed among the study population

Enamel Defect Type	Count	Percentage (%)
Demarcated opacities	816	49.28
Hypoplasia	408	24.64
Diffuse opacities	324	19.57
Demarcated opacity with hypoplasia	90	5.43
Other defects	6	0.36
Demarcated and diffuse opacities	6	0.36
Diffuse opacities with hypoplasia	6	0.36
Total enamel defects detected	1656	100

Further analysis of DDE severity demonstrated significant age-related differences across all tooth types. The distribution of DDE scores differed significantly among the three age groups for primary teeth (χ² = 120.657, p < 0.001), permanent molars (χ² = 53.636, p < 0.001), permanent canines (χ² = 33.369, p < 0.001), permanent premolars (χ² = 615.214, p < 0.001), and permanent incisors (χ² = 75.443, p < 0.001) (Table 5).

**Table 5:** Distribution of developmental defects of enamel (DDE) scores across age groups (3-6, 6-9, and 9-12 years) by tooth type — chi-square analysis

Parameters	DDE Score	3-6 Age Group	6-9 Age Group	9-12 Age Group	Chi-square	P-value
Primary tooth	0	6 (50.0%)	258 (91.5%)	870 (95.2%)	120.66	0.001
	1	0 (0.0%)	0 (0.0%)	14 (1.5%)		
	2	0 (0.0%)	0 (0.0%)	12 (1.3%)		
	3	6 (50.0%)	24 (8.5%)	12 (1.3%)		
	6	0 (0.0%)	0 (0.0%)	6 (0.7%)		
Permanent molars	0	6 (50.0%)	192 (68.1%)	630 (68.9%)	53.636	0.001
	1	6 (50.0%)	24 (8.5%)	68 (7.4%)		
	2	0 (0.0%)	0 (0.0%)	54 (5.9%)		
	3	0 (0.0%)	60 (21.3%)	156 (17.1%)		
	6	0 (0.0%)	6 (2.1%)	6 (0.7%)		
Permanent canines	0	12 (100.0%)	276 (97.9%)	872 (95.4%)	33.369	0.001
	1	0 (0.0%)	0 (0.0%)	36 (3.9%)		
	2	0 (0.0%)	0 (0.0%)	6 (0.7%)		
	3	0 (0.0%)	6 (2.1%)	0 (0.0%)		
Permanent pre-molars	0	0 (0.0%)	276 (97.9%)	842 (92.1%)	615.21	0.001
	1	0 (0.0%)	0 (0.0%)	30 (3.3%)		
	2	0 (0.0%)	0 (0.0%)	6 (0.7%)		
	3	0 (0.0%)	6 (2.1%)	36 (3.9%)		
	6	0(00.0%)	0 (0.0%)	0 (0.0%)		
Permanent incisors	0	6 (50.0%)	42 (14.9%)	90 (9.8%)	75.443	0.001
	1	6 (50.0%)	138 (48.9%)	494 (54.0%)		
	2	0 (0.0%)	54 (19.1%)	192 (21.0%)		
	3	0 (0.0%)	12 (4.3%)	90 (9.8%)		
	4	0 (0.0%)	0 (0.0%)	6 (0.7%)		
	5	0 (0.0%)	6 (2.1%)	0 (0.0%)		
	6	0 (0.0%)	30 (10.6%)	36 (3.9%)		
	7	0 (0.0%)	0 (0.0%)	6 (0.7%)		

Pairwise comparisons of DDE scores between age groups were performed using the Mann-Whitney U test with Holm adjustment for multiple comparisons. Significant differences were observed for primary teeth between all age-group comparisons (all adjusted p ≤ 0.014). No significant pairwise differences were observed for permanent molars following Holm correction (all adjusted p > 0.05). Permanent incisors demonstrated significantly lower DDE scores in the 3-6-year age group than in the 6-9-year (adjusted p = 0.0017) and 9-12-year (adjusted p = 0.0004) age groups, whereas no significant difference was observed between the 6-9-year and 9-12-year groups (adjusted p = 0.671). Permanent canines showed no statistically significant pairwise differences after Holm adjustment (all adjusted p > 0.05). For permanent premolars, a significant difference was observed between the 6-9-year and 9-12-year age groups (adjusted p = 0.0008) (Table 6).

**Table 6:** Pairwise Mann–Whitney between age bands (Holm-adjusted, per tooth type) NS = Not significant; * = Significant; ** = Highly significant.

Tooth Type	Comparison	N1	N2	Median 1	Median 2	p (raw)	p (Holm)
Primary teeth	3-6 vs 6-9	12	282	1.5	0	0	0.0000 (**)
	3-6 vs 9-12	12	914	1.5	0	0	0.0000 (**)
	6-9 vs 9-12	282	914	0	0	0.0139	0.0139 (*)
Permanent molars	3-6 vs 6-9	12	282	0.5	0	0.6525	0.6525 (NS)
	3-6 vs 9-12	12	914	0.5	0	0.6053	1.0000 (NS)
	6-9 vs 9-12	282	914	0	0	0.4984	1.0000 (NS)
Permanent incisors	3-6 vs 6-9	12	282	0.5	1	0.0008	0.0017 (*)
	3-6 vs 9-12	12	914	0.5	1	0.0001	0.0004 (**)
	6-9 vs 9-12	282	914	1	1	0.6714	0.6714 (NS)
Permanent canines	3-6 vs 6-9	12	282	0	0	0.6153	0.6153 (NS)
	3-6 vs 9-12	12	914	0	0	0.4485	0.8969 (NS)
	6-9 vs 9-12	282	914	0	0	0.0765	0.2296 (NS)
Permanent pre-molars	3-6 vs 6-9	12	282	3	0	0	0.0000 (**)
	3-6 vs 9-12	12	914	3	0	0	0.0000 (**)
	6-9 vs 9-12	282	914	0	0	0.0008	0.0008 (**)

Binary logistic regression analysis was performed to evaluate the association between age and the presence of DDE in the permanent dentition. Age, entered as a continuous variable, was a significant predictor of permanent-tooth DDE (B = 0.505, odds ratio = 1.657, 95% confidence interval: 1.410-1.947, p < 0.001). Each additional year of age was associated with a 65.7% increase in the odds of presenting with at least one developmental defect of enamel in the permanent dentition. The overall regression model was statistically significant (likelihood ratio test, p < 0.001) and correctly classified 96.0% of the study participants (Table 7).

**Table 7:** Binary logistic regression: age (years) as predictor of DDE on the permanent dentition DDE: Developmental defects of enamel. * = Significant.

Explanatory Variable	B	OR	p-value	95% CI	Tests of Model Coefficients (p)	Hosmer-Lemeshow Test (p)	% Correct Classification
Constant	-1.276		0.065		<0.001	0.000	96.0%
Age (years)	0.505	1.657	<0.001*	1.41–1.947			

## Discussion

To the best of our knowledge, this is the first epidemiological study evaluating DDE among the pediatric population of Kashmir using the modified DDE Index. The study was conducted at the only tertiary care dental center in Kashmir, which serves as the principal referral center for pediatric dental conditions in the region. Consequently, the majority of children with DDE are likely to present to this institution, making the study population broadly representative of the pediatric population seeking specialized dental care across Kashmir.

MIH is a developmental enamel defect involving the permanent first molars and, in some cases, the permanent incisors. It results from impaired enamel mineralization and presents clinically as well-defined opacities ranging in color from creamy white to yellow or brown. The severity and distribution of the lesions may differ between affected teeth. Because the hypomineralized enamel is structurally weak, it is more susceptible to post-eruptive breakdown and fracture under normal chewing forces. The etiology of MIH is considered complex and multifactorial, involving interactions between genetic predisposition and environmental influences. Prenatal, perinatal, and postnatal factors have been implicated, including maternal health complications during pregnancy, low birth weight, systemic illnesses during early childhood, episodes of high fever, and antibiotic exposure during the first years of life [9].

The findings of the present study demonstrate that DDE is prevalent among Kashmiri children and represents an important oral health concern requiring early identification and preventive intervention. Among the 3,308 affected teeth identified, 42 (1.2%) belonged to the 3-6-year age group, 696 (21.03%) to the 6-9-year age group, and 2,570 (77.69%) to the 9-12-year age group. Statistical analysis revealed a significant association between age group and the distribution of affected teeth (χ² = 214.87, df = 2, p < 0.001; Cramér's V = 0.255). The higher frequency of DDE in older children is clinically expected because a greater number of permanent teeth have erupted and become available for examination. Furthermore, enamel defects that originate during early childhood may only become clinically detectable after tooth eruption, resulting in a cumulative increase in prevalence with advancing age. Similar age-related trends have been reported by Robles et al. [10].

Permanent incisors constituted the majority of affected teeth (2,162; 65.35%), followed by permanent molars (754; 22.79%). A statistically significant association was observed between age group and tooth type affected (χ² = 250.78, df = 8, p < 0.001; Cramér's V = 0.195). The predominance of permanent incisors and molars may be attributed to the timing of amelogenesis and the increased susceptibility of these teeth to systemic disturbances during enamel formation. In addition, these teeth erupt early and are more frequently exposed to environmental and nutritional insults during critical developmental periods. The amelogenesis of permanent incisors and first molars occurs during early childhood, a developmental period characterized by vulnerability to febrile illnesses, nutritional deficiencies, and other systemic stressors. These findings support previous observations reported by Hasan et al. [11].

The prevalence of DDE in permanent dentition was substantially higher than that in primary dentition, with 1,160 (11.5%) affected permanent teeth compared with 68 (0.67%) affected primary teeth. A highly significant association was observed between dentition type and the occurrence of DDE (p < 0.001). Permanent teeth accounted for 95.04% of all affected teeth, whereas primary teeth constituted only 4.95%. This difference may be explained by the longer period of enamel formation in permanent teeth, which extends from birth through early childhood and increases susceptibility to systemic disturbances. Similar findings were reported by Robles et al., who observed a higher prevalence of DDE in permanent dentition than in primary dentition [10].

The predominance of DEO observed in the present study is consistent with previous literature. DEO accounted for 816 (49.28%) of all enamel defects, followed by hypoplasia (24.64%) and DIO (19.57%). The distribution of defect types was statistically significant (p < 0.001), indicating that enamel defects did not occur with equal frequency. The predominance of DEO suggests that localized disturbances affecting enamel mineralization during the maturation stage of amelogenesis may be more common than disturbances resulting in quantitative enamel loss. Hasan et al. similarly reported DEO as the most common defect type (28.76%), whereas combinations of DIO and hypoplasia were the least common [11]. Likewise, Olczak-Kowalczyk et al. found DEO to be the predominant enamel defect, followed by DIO and hypoplasia [12]. These findings reinforce the observation that qualitative enamel defects associated with hypomineralization are more prevalent than quantitative defects across different pediatric populations.

A cross-sectional study conducted by Palottil et al. among children aged 12-15 years reported an overall DDE prevalence of 20.7%, which was higher than that observed in the present study [13]. Their distribution of defect types, with DEO being the most common, corresponded closely with the current findings. Variations in prevalence between studies may be attributed to differences in geographic location, environmental exposure, nutritional status, socioeconomic conditions, sample size, age groups studied, and diagnostic criteria employed. Similarly, Disha et al. reported a prevalence of enamel defects of 12.8% among children aged 8-12 years, highlighting that developmental enamel defects continue to represent an important public health concern [14].

Although the prevalence observed in the present study was comparatively lower than that reported in some previous cohorts, the findings remain clinically significant because DDE can adversely affect esthetics, mastication, and oral health-related quality of life. Furthermore, enamel defects predispose affected teeth to hypersensitivity, plaque accumulation, post-eruptive enamel breakdown, and increased susceptibility to dental caries. Hypomineralized enamel possesses compromised mechanical properties, which may negatively affect the longevity and retention of restorative treatments.

Binary logistic regression demonstrated that increasing age was independently associated with the presence of DDE in the permanent dentition, with each additional year increasing the odds of permanent-tooth DDE by approximately 66%. This association is biologically plausible because the eruption of permanent teeth with advancing age increases the number of enamel surfaces available for examination, allowing developmental defects to become more readily detectable. Pairwise comparisons further supported this finding, showing a progressive decline in DDE scores in the primary dentition with age, while permanent molars and canines remained relatively stable across age groups. Permanent incisors showed significant differences only between the youngest and older age groups, reflecting the mixed dentition stage rather than progressive worsening of defects. Overall, these findings suggest that the age-related pattern of DDE is influenced by both the timing of tooth eruption and the physiological transition from primary to permanent dentition. The present study found a significant association between age and the distribution of DDE across all tooth types (Chi-square, p < 0.001). The observed pattern is consistent with normal tooth eruption and exfoliation, with defects in primary teeth decreasing with age and DDE in permanent teeth becoming more evident as they erupt. Permanent incisors showed the greatest variation in DDE scores, while permanent molars consistently exhibited moderate defects. In contrast, permanent canines and premolars were largely unaffected, reflecting their later eruption. These findings suggest that age and tooth eruption stage play an important role in the clinical detection and distribution of DDE and support the importance of screening during the mixed dentition period.

The present study has certain limitations. Being hospital-based, the study population may not be fully representative of the general pediatric population, introducing the possibility of selection bias. In addition, although standardized diagnostic criteria and examiner calibration were employed, measurement bias and inter-examiner variability cannot be completely excluded. Furthermore, systemic, prenatal, perinatal, nutritional, genetic, and environmental risk factors associated with DDE were not evaluated and therefore could not be examined as potential confounding variables. Another important consideration is that the large sample size may have increased the likelihood of detecting statistically significant associations despite relatively small effect sizes. Chi-square analysis does not control for potential confounding factors. However, due to the cross-sectional nature of the study and limitations in the available dataset, multivariable logistic regression analysis was not performed.

Sex-specific data were not available for analysis in the present study. We acknowledge that sex may be an important confounding variable. Future investigations should collect and analyze sex-disaggregated data to better understand its potential influence on DDE prevalence and distribution.

Despite these limitations, the present study provides important baseline epidemiological data regarding DDE among Kashmiri children. Early diagnosis and timely management of enamel defects are essential to minimize functional and esthetic complications. Preventive and treatment-oriented oral health programs targeting schoolchildren should be implemented to improve awareness regarding DDE and promote early dental intervention. Long-term follow-up of affected children is also recommended to reduce the risk of dental caries, hypersensitivity, and restorative failure associated with defective enamel.

## Conclusions

The present study demonstrated a considerable prevalence of DDE among children in the Kashmiri pediatric population. Permanent incisors and molars were the most frequently affected teeth, with DEO being the predominant type of enamel defect observed. The higher prevalence of DDE in older children and in the permanent dentition emphasizes the importance of early detection and routine dental screening during childhood. DDE may significantly compromise esthetics, tooth sensitivity, and overall oral health while also increasing the risk of dental caries and restorative challenges. Therefore, timely diagnosis and preventive intervention are essential to minimize long-term complications and improve functional and psychological outcomes in affected children.

The findings of the present study provide baseline epidemiological data for the Kashmiri population and underscore the need for further multicentric and community-based studies to investigate the etiological factors, risk determinants, and long-term outcomes associated with DDE. However, as the findings are based on a specific sample and study setting, caution should be exercised when extrapolating these results to other populations. Further multicenter studies with representative samples are needed to confirm these findings. In addition, awareness programs for parents, school-based oral health initiatives, and preventive treatment protocols should be encouraged to promote early management of enamel defects and improve pediatric oral health outcomes.
